# Genomic analyses of *Staphylococcus aureus* clonal complex 45 isolates does not distinguish nasal carriage from bacteraemia

**DOI:** 10.1099/mgen.0.000403

**Published:** 2020-07-15

**Authors:** Chandler Roe, Marc Stegger, Berit Lilje, Thor Bech Johannesen, Kim Lee Ng, Raphael N. Sieber, Elizabeth Driebe, David M. Engelthaler, Paal Skytt Andersen

**Affiliations:** ^1^​ Translational Genomics Research Institute, Flagstaff, AZ, USA; ^2^​ Northern Arizona University, Flagstaff, AZ, USA; ^3^​ Bacteria, Parasites and Fungi, Statens Serum Institut, Copenhagen, Denmark; ^4^​ Department of Veterinary and Animal Sciences, University of Copenhagen, Frederiksberg, Denmark

**Keywords:** genome-wide association, *Staphylococcus aureus*, bacteraemia, nasal carriage, CC45

## Abstract

*
Staphylococcus aureus* is a colonizing opportunistic pathogen and a leading cause of bloodstream infection with high morbidity and mortality. *S. aureus* carriage frequency is reportedly between 20 and 40 % among healthy adults, with *S. aureus* colonization considered to be a risk factor for *S. aureus* bacteraemia. It is unknown whether a genetic component of the bacterium is associated with *S. aureus* bacteraemia in comparison to nasal carriage strains. Previous association studies primarily focusing on the clinical outcome of an *S. aureus* infection have produced conflicting results, often limited by study design challenged by sample collections and the clonal diversity of *S. aureus *. To date, no study has investigated whether genomic features separate nasal carriage isolates from *S. aureus* bacteraemia isolates within a single clonal lineage. Here we have investigated whether genomic features, including single-nucleotide polymorphisms (SNPs), genes, or kmers, distinguish *S. aureus* nasal carriage isolates from bacteraemia isolates that all belong to the same clonal lineage [clonal complex 45 (CC45)] using whole-genome sequencing (WGS) and a genome-wide association (GWA) approach. From CC45, 100 isolates (50 bacteraemia and 50 nasal carriage, geographically and temporally matched) from Denmark were whole-genome sequenced and subjected to GWA analyses involving gene copy number variation, SNPs, gene content, kmers and gene combinations, while correcting for lineage effects. No statistically significant association involving SNPs, specific genes, gene variants, gene copy number variation, or a combination of genes was identified that could distinguish bacteraemia isolates from nasal carriage isolates. The presented results suggest that all *S. aureus* nasal CC45 isolates carry the potential to cause invasive disease, as no core or accessory genome content or variations were statistically associated with invasiveness.

## Data Summary 

Read data were deposited in the Sequence Read Archive (SRA) under BioProject PRJNA417115 (https://www.ncbi.nlm.nih.gov/bioproject/PRJNA417115).

**Impact Statement**

Here, we analysed *Staphylococcus aureus* CC45 genomes from 50 nasal carriage isolates and 50 bacteraemia isolates collected in 2 major cities in Denmark. Our aim for this study was to investigate possible genetic associations with either phenotype. Previous studies investigating these associations have reported study limitations caused by multiple factors, including limited informatic tools as well as the inclusion of multiple clonal complexes, which complicated potential findings. Furthermore, multiple studies have reported conflicting results. Our study applies extensive bioinformatic analyses performed on a single clonal complex, which allowed for a less biased, more focused approach. The findings presented here indicate that there are no statistically significant genetic associations that can differentiate nasal carriage *S. aureus* from bacteraemia *S. aureus *, suggesting that all S. aureus nasal carriage CC45 isolates carry the potential to cause invasive disease.

## Introduction

*Staphylococcus aureus* is a Gram-positive colonizing opportunistic pathogen capable of causing a wide spectrum of infections and is the second most frequent pathogen responsible for bacteraemia. *S. aureus* bacteraemia (SAB) has a reported incidence rate averaging 25 per 100 000 persons annually in both North American and Western European countries [1]. SAB results in significant morbidity and mortality with estimated mortality rates of 10–30 % [2] and is responsible for causing more patient deaths than *Streptococcus pneumoniae *, *Neisseria meningitidis *, *Haemophilus influenzae* and *Streptococcus pyogenes* combined [3]. A major risk factor associated with SAB is *S. aureus* colonization; studies have demonstrated *S. aureus* colonization rates of as high as 40 % in the adult population [4, 5]. Using pulsed-field gel electrophoresis (PFGE), a correlation has been demonstrated between nasal carriage isolates and the corresponding bloodstream isolate in 80 % of cases when nasal isolates were obtained prior to SAB infections [4]. Despite high *S. aureus* colonization rates among the general population and the associated risk of the development of SAB, the majority of *S. aureus *-colonized individuals will remain unaffected by their commensal *S. aureus* isolates [6]. However, previous research has demonstrated an overlap between *S. aureus* nasal carriage lineages and bacteraemia-causing lineages [4]. Specifically, *S. aureus* lineage CC45 is one of the most prevalent lineages in both nasal colonization and bloodstream infections [7–9].

To date, there is limited understanding of the transition from commensal *S. aureus* colonization to SAB [10]. Reported host risk factors for the development of invasive *S. aureus* infections include age, ethnicity, end-stage renal disease, chronic wounds and HIV status [6, 11]. While several known host risk factors exist, research investigating nasal carriage strain progression to invasive disease has been limited by study design and sample sets [6, 10, 12]. Genome-wide association (GWA) studies have been applied in an effort to identify the genetic variants responsible for host susceptibility to *S. aureus* infections [13, 14]. A recent study investigating genetic differences between SAB and *S. aureus* endocarditis isolates was unable to genetically distinguish isolates from either infection type, suggesting that there is not a specific *S. aureus* genotype associated with infective endocarditis when a patient has SAB [15]. Furthermore, an additional study reported similar results, with no specific genotypes associated with methicillin-sensitive *S. aureus* (MSSA) endocarditis or MSSA bacteraemia [16]. While these two studies are in agreement with both subject and results, several studies have compared varying clinical patient groups, applied contrasting methods and produced inconsistent results [15]. Multiple studies have investigated clonal complex 30 (CC30) and an association with endocarditis; three studies found no association [16–18], two studies reported a link to CC30 and increased invasive disease [19, 20], and a final study found CC30 to be associated with nasal carriage only [21]. While these studies addressed whether specific genotypes are associated with clinical manifestations, few studies have investigated potential genetic components or variants correlated with invasive disease progression from a single *S. aureus* sequence type. A recent study investigated the genomic evolution of *S. aureus* nasal isolates progression to bacteraemia, but with only eight patients in the study, statistical inference was limited [6]. Here we have investigated whether genomic features, including single-nucleotide polymorphisms (SNPs), genes, or kmers, distinguish *S. aureus* nasal carriage isolates from bacteraemia isolates that all belonged to clonal lineage (CC45) using whole-genome sequencing (WGS) and a GWA approach. By using a single-lineage approach, we strengthened our study by reducing the genomic noise that may occur in GWA studies with multiple lineages.

## Methods

### Isolate collection

A total of 100 methicillin-susceptible *S. aureus* CC45 isolates were used in this study. Samples fell into 2 categories: 50 isolates were identified as the causative agent of bacteraemia confirmed by positive blood cultures and 50 isolates were isolated from nasal swabs. The samples from the two groups are referred to as SAB (* S. aureus* bacteraemia) and NC (nasal carriage) isolates, respectively. Both SAB and NC samples were collected in 2009 from two regions in Denmark, Copenhagen and Aarhus (Table S1, available in the online version of this article).

### DNA sequencing, assembly and multilocus sequence typing (MLST) typing

Blood samples from patients were plated and single colonies picked and stored at −80 °C until processing. Samples were grown on trypticase soy agar (TSA) (BD, Franklin Lakes, USA) at 37 °C for 24 h, after which DNA was extracted using the Qiagen DNeasy Blood and Tissue Purification kit including a pre-lysis step using lysostaphin for Gram-positive extractions according to the manufacturer’s recommendations (Qiagen, Valencia, CA, USA). DNA was prepared for multiplexed, paired-end sequencing (2×100 bp) on an Illumina GA*_IIX_* instrument using the Library Preparation kit with standard PCR library amplification (KAPA Biosystems, Woburn, MA, USA) as described previously [22]. Additionally, six samples that failed initial sequencing were resequenced on an Illumina MiSeq instrument using 2×250 V2 technology (Table S1). The average coverage across all 100 isolates was 130×. Genomes were assembled with UGAP (https://github.com/jasonsahl/UGAP), which utilizes SPAdes v3.10.1 [23] and Pilon [24] post-assembly error correction. MLST was performed with the raw reads using SRST2 [25].

### SNP detection

In order to infer genomic relatedness, a high-quality core SNP matrix was generated using the pipeline NASP v1.0 [26]. Briefly, raw reads were aligned to the publicly available ST45 reference chromosome CA-347 (GenBank accession number CP006044) [27] using the Burrows–Wheeler aligner (BWA) v0.7.7 [28]. The program NUCmer was used to identify duplicate regions within the reference genome [29] and those positions were subsequently removed from the analysis. SNPs were identified using the unified genotyper within the Genome Analysis Toolkit (GATK) [30] v3.3.0. SNP loci were only retained in the final dataset if the position was present in every sample with at least 10× coverage and the allele per isolate had a proportion of >90 % of the reads. Nucleotide substitution model testing was performed within the program IQ-TREE [31] v1.6.1. A maximum-likelihood whole-genome SNP phylogeny was produced using IQ-TREE and the determined best nucleotide model with 1000 pseudoreplicate bootstrapping. The tree was visualized in FigTree v1.4.3 (http://tree.bio.ed.ac.uk/software/figtree/). For downstream SNP analyses, the SNP matrix data was transformed; bases identical to the reference genome were coded as ‘0’, while any base different from the reference was coded as ‘1’ [approximately 30 SNP loci (0.26 %) had more than 2 observed allele states, but all variants were coded as ‘1’]. The R package ‘Differential Analysis of Principal Components’ (DAPC) [32] using adegenet [33] v2.1.1 was used to perform multivariate comparisons on the SNP matrix. The optimal number of principal components (PCs) required for DAPC was identified by performing multiple cross-validations using the xvalDapc function [15]. If an optimal number of PCs was not identified, further analysis was terminated.

### SNP density

The accumulation of SNPs within coding regions was examined using the Tool for Rapid Annotation of Microbial SNPs (TRAMS) software v1.0.2 with default settings [34]. Additionally, TRAMS identified whether each SNP was synonymous or nonsynonymous. In order to examine SNP density within non-coding regions, a 1000 bp sliding window with an increment of 1 bp was implemented using the master matrix produced in NASP. Within each sliding window, the number of SNPs within each sample was calculated. The numbers of SNPs and SNP distributions were then compared between the two groups, NC and SAB. In an effort to reduce the number of statistical tests performed on the SNP densities, windows were only assessed when the log_2_FC (log_2_ of fold changes) between the mean number of SNPs in the two groups was >0.1 or <−0.1. Similarly, only unique SNP combinations (excluding singletons) within NC or SAB, respectively, were calculated and corrected for multiple testing. SNP patterns that would yield significant *P* values after multiple testing corrections can be viewed in Fig. S1. Based on this analysis, a SNP present in 31 of 50 (62 %) NC isolates and 8 of 50 (16 %) SAB isolates would show a significant difference (*P* value <0.05). Another example of a significant combination of isolates containing a SNP is 22 of 50 (44 %) SAB isolates and 3 of 50 (6 %) NC isolates. This would also yield a *P* value below 0.05 after Bonferroni correction.

### Accessory genome

Using the UGAP assemblies for each sample, open reading frames were identified and annotated using the default settings for the program Prokka v1.2 [35]. Using the Prokka results, gene presence/absence for all samples was defined with the program Roary v3.6.0 [36]. Additional multivariate analyses were performed as described above. If any sequences were found to belong to only one group, the sequence was extracted and megablast v2.2.29 [37] and tblastx v2.2.29 [38] were used to confirm the results. Additionally, VirulenceFinder v2.0 [39] was used to identify known virulence factors within the database and these were investigated for correlation with SAB.

### GWA using the accessory genome

An additional GWA analysis was conducted using the following approach. First, raw reads were aligned to the pangenome reference output file produced from Roary [36] v3.11.0 using blastp [38] in order to identify the accessory genome. Using the program Mykrobe [40], raw reads from all isolates were tested against a panel of all genes identified by Roary. A gene was considered present with a gene coverage ≥80 % and a median sequencing depth of at least 5. Simultaneously, a pairwise blastn [38] of all versus all accessory genes using a minimum identity of 99 % and length of >20 % generated a matrix identifying duplicated genes within the dataset and these were removed from downstream analyses. Additionally, genes with a prevalence of <5 % or >95 % were removed. These combined results were used as input for the R-based package treeWAS v1.0 [41] to determine accessory gene association with SAB and NC isolate sources. For further investigation into the association with phenotype, we also applied additional GWA software, pySEER [42]. Briefly, raw sequencing reads were fragmented into 54 mers, which were tested for association with either phenotype. In order to account for population structure, a similarity matrix was included. pySEER was run using default settings and a maf value of 0.05. Output was visualized using ggplot2 v2.1.0 in R software v3.4.2 [43].

### Gene copy number variation detection

Copy number variation was examined using the program CNOGpro [44] v1.1. First, raw reads were aligned to the *S. aureus* CA-347 reference chromosome using BWA. Using the .bam file, a list containing the chromosome identifier as well as each aligned reads’ leftmost coordinate was created and used as input into CNOGpro, along with a GenBank file of the reference chromosome. CNOGpro was implemented in R with default parameters and a window length of 100 bases.

### Kmer analysis and statistics

The assembled genomes were fragmented (kmer size: 30 bp), and kmers were added to a python dictionary as described previously [15]. The number of occurrences for each kmer or reverse complement of the kmer was recorded. In order to account for long kmer repeats skewing the kmer counts, each kmer was only counted once per sample. Additionally, our analysis did not allow kmers to span contig junctions. The presence/absence of unique kmers was compared for each group. Statistical analyses were conducted using R software v3.4.2. Distribution comparisons were implemented using the Mann–Whitney U test and proportion tests were performed using Fisher’s exact test. Multiple testing was corrected for using the false discovery rate (FDR) method with a significance level of 0.05. The R package ggplot2 v2.1.0 was used to generate visualizations unless otherwise stated [45]. In order to improve statistical power to detect informative kmers, all unique kmers combinations (excluding singletons) were calculated and corrected for multiple testing. An example of a kmer pattern that would yield a significant *P* value after multiple testing correction using Bonferroni are kmers present within 33 or more of the 50 NC isolates and 8 or fewer of the 50 SAB isolates (as shown for SNPs in Fig. S1).

## Results

### GWA investigation

**Fig. 1.**
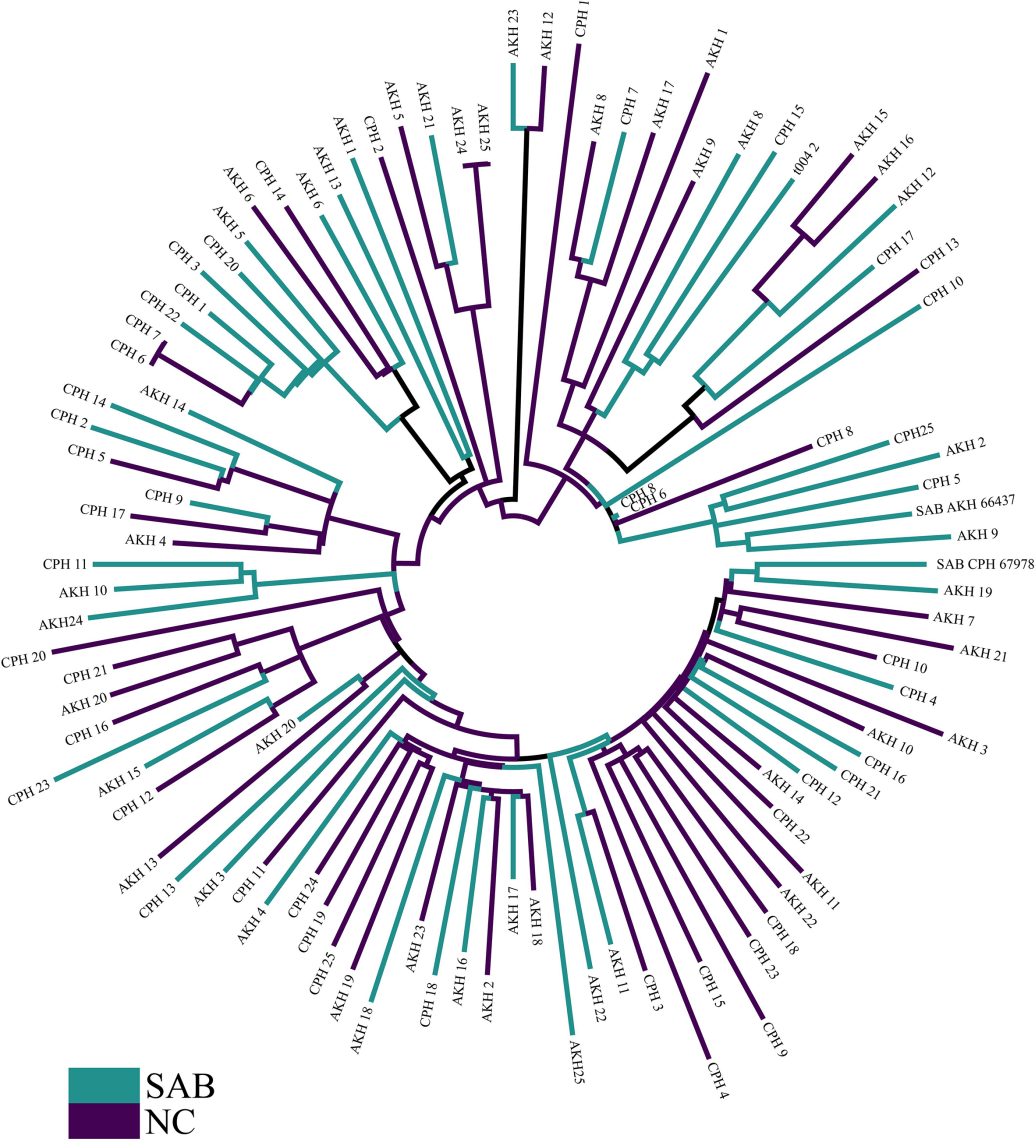
Rooted maximum-likelihood phylogeny produced from 12 064 SNPs detected within the core genome of 100 contemporary isolates from Denmark. Nasal carriage samples are in purple while bacteraemia samples are in green.

**Fig. 2.**
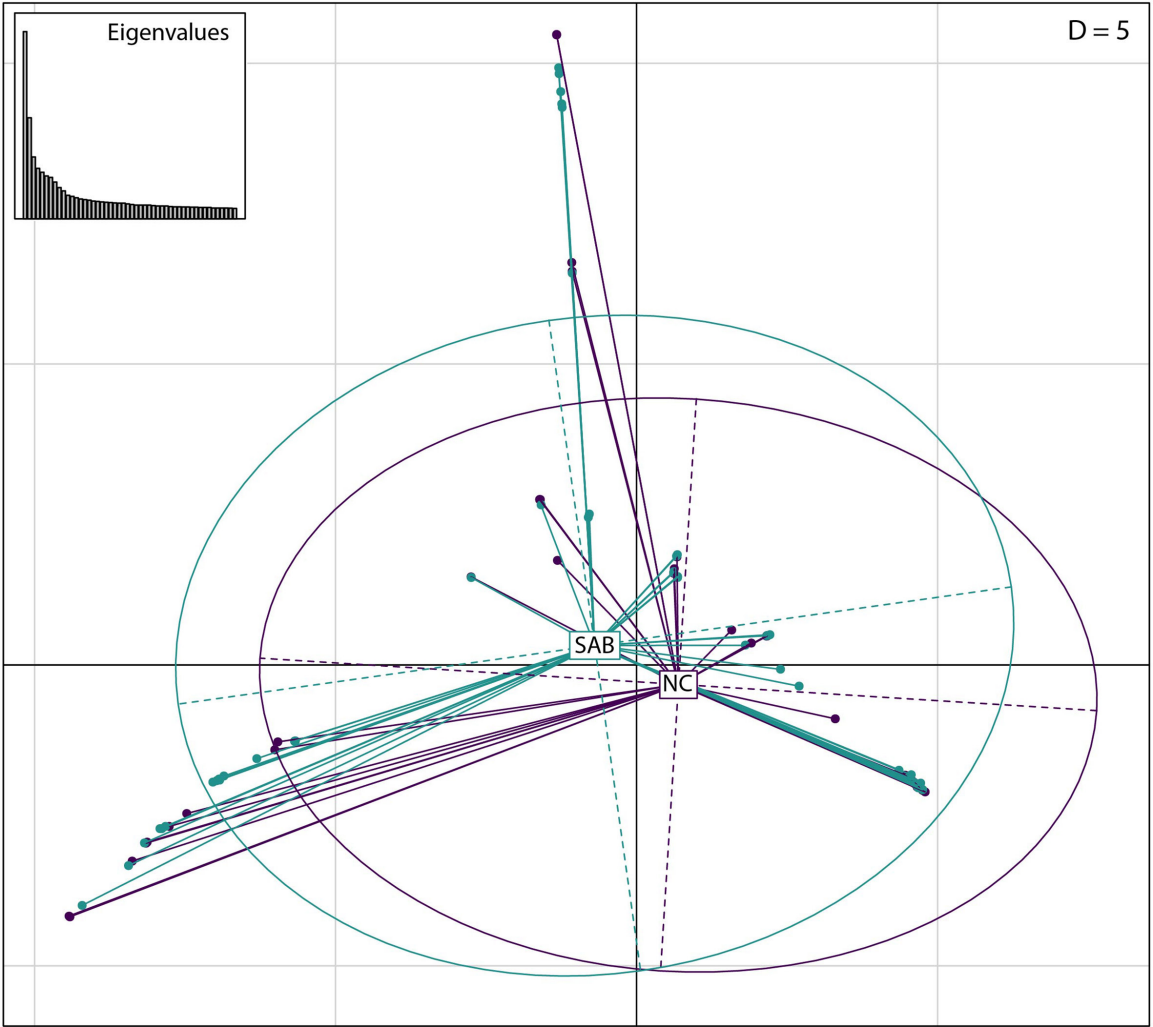
PCA plots demonstrate the relatedness of 50 NC isolates with 50 SAB isolates. The dudi.pca function in R was used to generate this PCA plot in which two axes were retained. Samples are coloured by source type; SAB is green while NC isolates are purple. Samples clustered randomly rather than by infection type.

**Fig. 3.**
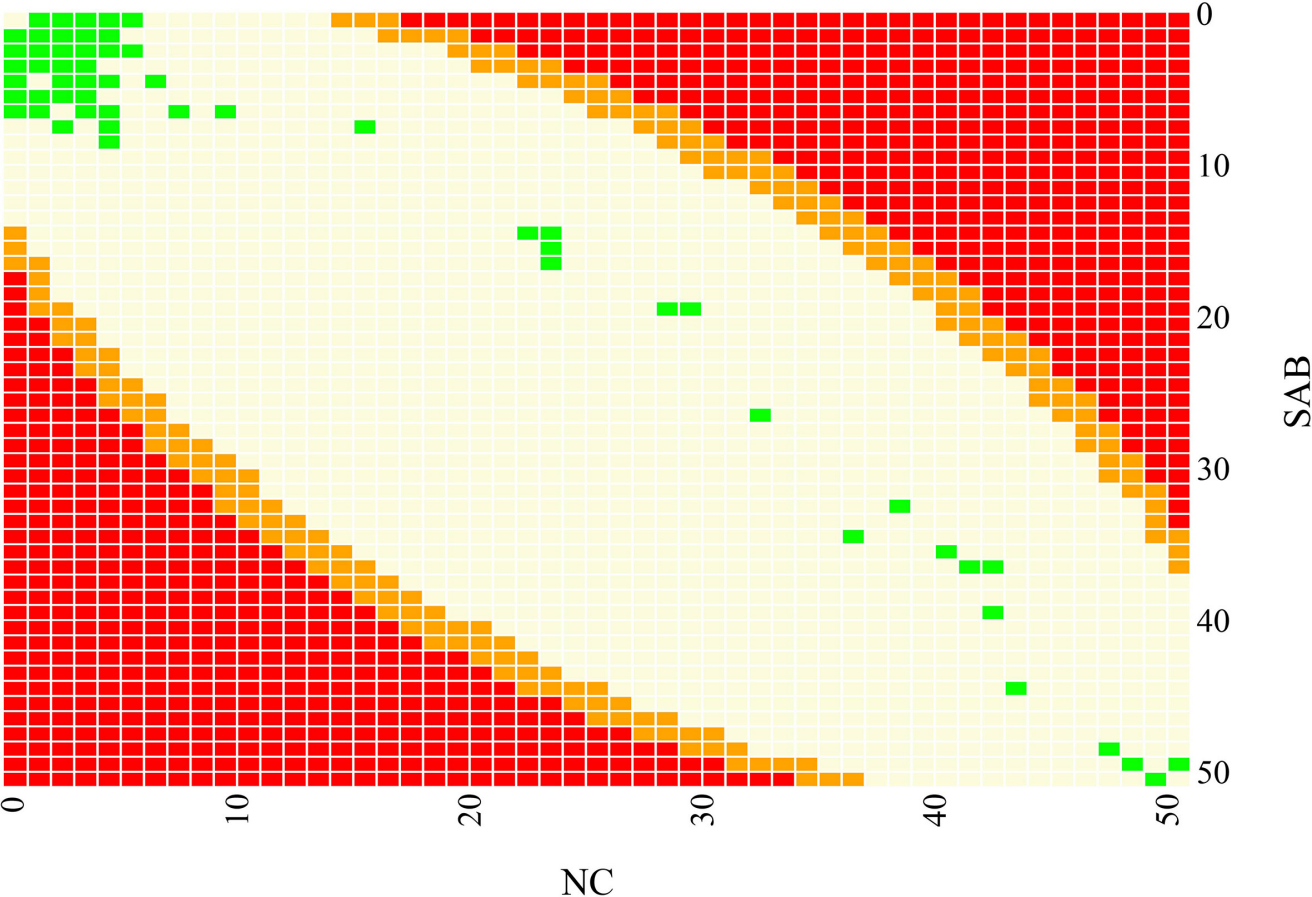
SNP significance level in comparison to SNP distribution. Each colour represents varying significance values after multiple testing correction; red represents a *P* value <0.05, orange represents a *P* value >0.05 and <1, and light yellow represents a *P* value=1. All 12 064 SNPs from this dataset are marked in green. This figure demonstrates that none of the SNPs identified in this study were significantly associated with either SAB or NC infection type.

We sequenced the genomes of 100 *S *. *aureus* CC45 samples collected from contemporary Danish bacteraemia and nasal swabs, and genomic analysis and molecular typing revealed that all isolates were MSSA and ST45. The sequenced isolates were defined as two groups, NC and SAB, based on isolate source location. In total, the NASP pipeline identified 12 064 high-quality SNPs in more than 1.9 Mb of the reference genome that were subsequently examined to differentiate nasal carriage from bacteraemia samples. Of the 12 064 SNPs, 85 % (10 244) were autapomorphic. A maximum-likelihood phylogenetic tree, using the TVMe +ASC nucleotide substitution model, did not identify any major clustering of SAB samples or NC samples (Fig. 1). Similarly, no significant clustering was identified by principle component analysis (PCA) among stratified groups (Fig. 2). No single SNP demonstrated statistical over-representation in either group, when individual SNP positions were assessed for differentiation between NC from SAB using R (Fig. 3). None of the tested PCs predicted NC vs SAB better than by random chance using DAPC, indicating that no such signal was present within the dataset.

A gene prone to high SNP accumulation could potentially distinguish the two groups (e.g. the proposed accessory gene regulator (*agr*) gene [6]); however, different mutations within such a gene would likely occur in separate samples, and so additional methods are required to identify this distinction. TRAMS, using the annotated reference strain CA-347, was used to calculate the SNP accumulation in 1000 bp windows across all samples. SNP densities were then compared among NC and SAB isolates. No association of SNP density within predicted coding regions was observed for either NC or SAB isolates (FDR-adjusted *P* values >0.9677). SNP accumulation within non-coding and non-annotated regions was also investigated using a 1000 bp sliding window approach with the NASP-generated SNP matrix. Again, no SNP accumulations differentiating SAB from NC were identified in these regions (FDR-adjusted *P* values >0.2838).

### Mutations in non-core genome

The approach outlined above investigated SNPs identified within the high-quality core genome, but would not identify distinguishing mutations within the non-core genome. In order to determine whether mutations within the accessory genomes as defined by the *S. aureus* reference chromosome CA-347 were associated with NC or SAB phenotypes, we extracted the nucleotide calls for all positions for all isolates as provided by the NASP pipeline as a master position matrix. We found no association of uncalled positions (including invariant positions) between the two groups of interest. Further, no significant differences were identified in the proportions of SNP, non-SNP and uncalled positions between phenotypes (FDR-adjusted *P* values >0.283).

### Gene presence/absence

As increased virulence is often associated with specific genetic profiles, we examined the genetic content for virulence factors as well as gene content association between NC and SAB using our assembled genomes and the programs Prokka [35] and Roary [36]. No genes were significantly associated with either NC or SAB. No positive signal was identified when using DAPC to examine whether gene combinations could distinguish bacteraemia samples from nasal carriage samples. The genomes were also screened for the presence/absence of known virulence-related genes for each isolate. Of the 1465 reported virulence genes in the virulenceFinder database that were screened for, we did not identify any virulence content that differentiated SAB from NC isolates (Fig. S2).

### Kmer over-representation

While a variety of tools were used to identify SNPs or genes associated with either NC or SAB, these methods depend on either a reference sequence or accurate gene predictions. In order to identify any genomic differences between NC and SAB isolates without these previous assumptions, we examined all DNA sequences from the *de novo* assemblies for sequence over-representation in either group using a kmer approach. All assemblies were fragmented into overlapping 30 bp kmers and the number of times each kmer (or its reverse-complement) appeared in every sample was documented using a previously published script [15]. Unique kmers found only in single isolates were removed from the analysis. We observed no significant over-representation associated for either NC or SAB for any kmer in the dataset (FDR-adjusted *P* values >0.1055).

### Accessory genome association controlled for population structure

Using the pangenome reference produced by the program Roary, we determined gene presence/absence for all isolates and reduced the number of genes to obtain a better representation of true genetic content across the collection of samples using Mykrobe and treeWAS. Using this approach, we identified no accessory gene content that could distinguish SAB from NC isolates. Furthermore, 1617 kmers from raw sequencing reads were identified as significantly associated with 1 phenotype with pySEER. However, of these, 1610 were flagged with a bad chi-square indicating low sample numbers. The seven remaining kmers were mapped to the reference genome and investigated manually, which identified them as false positives originating from short repetitive regions.

### Copy number variation

Both copy number variation as well as gene copy number correlations between sample type were investigated, but all lacked the statistical power necessary to correlate the gene copy variation with either phenotype.

## Discussion

Bloodstream infections represent 15 % [46] of all nosocomial infections and are a major public health concern [47], with *S. aureus* reported to be the second most common cause of nosocomial bloodstream infections. Over a 12-year period within a single 900-bed tertiary care hospital in the USA, *S. aureus* accounted for 13 % of hospital-acquired bacteraemia [48]. A major risk factor associated with *S. aureus* bloodstream infections is *S. aureus* nasal carriage [4, 49]. Previous research has suggested that spontaneous mutations in the *S. aureus* colonizing strain of a patient supersede disease progression, but the circumstances leading to invasive disease are still not well understood [50, 51]. This study implemented whole-genome comparative analyses of both bacteraemia infections and nasal carriage isolates from a single *S. aureus* lineage in order to determine genomic features associated with either phenotype. Using GWA analyses, we examined individual SNPs, SNP combinations, the presence/absence of genes, gene copy number variation and unique kmers in an effort to genomically differentiate SAB from nasal carriage isolates and demonstrated that, at least for the CC45 lineage, *S. aureus* nasal carriage isolates are not genotypically distinct from the *S. aureus* isolates responsible for bacteraemia. However, this does not investigate the virulence of the CC45 lineage itself compared to other lineages of *S. aureus *.

While past studies have investigated the progression of *S. aureus* nasal colonization to bacteraemia, they have been hindered by sample sets, varying genetic methods and conflicting findings [6, 10]. In 2014, a study investigated whether genetic variants within the *S. aureus* genomes were associated with *S. aureus* bacteraemia using a GWA approach. That study compared 361 bacteraemia cases that spanned 14 different clonal complexes and was unable to identify variants associated with invasive disease progression. However, that study was further complicated by the inclusion of multiple genetic lineages [52]. Another investigation into the evolution of *S. aureus* during disease progression identified eight mutations associated with bacteraemia. While these results stemmed from a longitudinal study incorporating 169 isolates, the study only identified these mutations within 1 patient with a bloodstream infection, and thus lacked statistical support [10]. Interestingly, none of these eight mutations were present in our dataset. A more recent study found a mutation within the *agrA* gene causing a loss of function in correlation with bacteraemia progression in a single patient [6]. Our study investigated two outcomes for *S. aureus* (nasal carriage and bacteraemia) across a single lineage. Our comprehensive analyses of 100 contemporary and geographically matched CC45 *S. aureus* isolates did not reveal any genetic association between nasal carriage and bacteraemia isolates, suggesting that host susceptibility or environmental factors are likely more important for the development of invasive *S. aureus* disease. These results have implications within the healthcare field for infection prevention. Previous studies have demonstrated a decrease in the risk of development of a surgical site infection from *S. aureus* through perioperative decolonization of patients using intranasal mupirocin [53–55]. A more widespread implementation of decolonization of patients prior to invasive procedures may limit the number of cases of *S. aureus* bacteraemia.

A limitation of this study is that our samples were not longitudinally paired from 50 individual patients whose *S. aureus* nasal isolates progressed into bacteraemia infections. Instead, our samples were from 100 separate individuals, 50 of whom had bacteraemia infections and 50 of whom were nasally colonized with *S. aureus *. We therefore did not study the evolution of *S. aureus* within each patient during disease progression, which may overlook private mutations related to invasiveness. This has been done in a recent study of within-host evolution, where hotspots of parallel evolution during the transition from colonizing to invasive isolates were described [56]. These mutations may, however, be the result of invasiveness rather than the cause and our study showed that there is no general genotypic feature determining whether a *S. aureus* CC45 is prone to become invasive or not, but invasiveness rather seems to be a truly opportunistic process. Another limitation of our study is that we sequenced a single isolate from each patient, thus missing potential within-host diversity, and therefore, perhaps overlooking important adaptive traits. Furthermore, the current methods for copy number variation analysis using short-read data cannot easily capture repetitive regions or chromosomal rearrangements and this is a shortcoming of this study.

Despite these limitations, this study reports an important observation regarding *S. aureus* disease progression from nasal carriage. Using various GWA approaches that allowed for robust and comprehensive investigations of 100 *S *. *aureus* CC45 genomes, we demonstrated that the ability of *S. aureus* to cause bacteraemia is not associated with any identifiable genetic factors, including genes, SNP combinations and copy number variation. These results provide further support for the contention that it is more likely that host risk factors precipitate the onset of invasive *S. aureus* disease. This study showed that all *S. aureus* nasal CC45 isolates carry the potential to cause invasive disease.

## Supplementary Data

Supplementary material 1
