# Supplementary material for: Genomic analyses of Staphylococcus aureus clonal complex 45 isolates does not distinguish nasal carriage from bacteraemia
Source: Microb Genom. 2020 Jul 15;6(8):mgen000403. doi: 10.1099/mgen.0.000403 (PMC7641415; doi:10.1099/mgen.0.000403)
Supplement: Supplementary material 1 [file mgen-6-403-s001.pdf]

Supplemental Table S1. Isolate data including isolation location, sample type, average sequencing coverage, number of contigs, N50, largest contig, total length of assembled genome, and the sequencing platform.

Sample Name	Isolation Location	Sample Type	Average Sequencing Coverage	Number of Contigs	N50	Largest Contig	Total Length	Sequencing Platform
NC_AKH_10	Aarhus	Nasal Carriage	20	38	185631	491086	2733135	GAIIX
NC_AKH_11	Aarhus	Nasal Carriage	121	30	184586	468771	2714117	GAIIX
NC_AKH_12	Aarhus	Nasal Carriage	134	34	215464	649832	2687613	GAIIX
NC_AKH_13	Aarhus	Nasal Carriage	48	36	166743	386068	2730407	GAIIX
NC_AKH_14	Aarhus	Nasal Carriage	84	309	73603	334064	3032415	GAIIX
NC_AKH_15	Aarhus	Nasal Carriage	83	50	209961	551074	2769543	GAIIX
NC_AKH_16	Aarhus	Nasal Carriage	712	60	213811	697926	2764707	GAIIX
NC_AKH_17	Aarhus	Nasal Carriage	83	33	214924	664848	2707175	GAIIX
NC_AKH_18	Aarhus	Nasal Carriage	146	39	184680	678894	2676684	GAIIX
NC_AKH_19	Aarhus	Nasal Carriage	132	96	185546	568193	2736006	GAIIX
NC_AKH_1	Aarhus	Nasal Carriage	130	44	179416	510258	2758733	GAIIX
NC_AKH_20	Aarhus	Nasal Carriage	120	35	176479	316329	2711276	GAIIX
NC_AKH_21	Aarhus	Nasal Carriage	254	54	184674	313343	2703284	GAIIX
NC_AKH_22	Aarhus	Nasal Carriage	79	38	184704	648639	2721462	GAIIX
NC_AKH_23	Aarhus	Nasal Carriage	82	36	166400	329736	2682163	GAIIX
NC_AKH_24	Aarhus	Nasal Carriage	155	42	166876	554951	2772199	GAIIX
NC_AKH_25	Aarhus	Nasal Carriage	146	43	158298	326579	2778809	GAIIX
NC_AKH_2	Aarhus	Nasal Carriage	75	33	183954	494675	2695848	GAIIX
NC_AKH_3	Aarhus	Nasal Carriage	39	37	185718	269418	2718293	GAIIX
NC_AKH_4	Aarhus	Nasal Carriage	61	36	185721	446836	2726503	GAIIX
NC_AKH_5	Aarhus	Nasal Carriage	74	40	162471	460526	2747992	GAIIX
NC_AKH_6	Aarhus	Nasal Carriage	114	31	313407	495074	2734196	GAIIX
NC_AKH_7	Aarhus	Nasal Carriage	52	26	195438	676623	2712434	GAIIX
NC_AKH_8	Aarhus	Nasal Carriage	43	32	185592	384346	2648943	GAIIX
NC_AKH_9	Aarhus	Nasal Carriage	202	26	212173	380738	2738167	GAIIX
NC_CPH_10	Copenhagen	Nasal Carriage	91	36	173089	494291	2697086	GAIIX
NC_CPH_11	Copenhagen	Nasal Carriage	18	37	124561	409160	2705946	GAIIX
NC_CPH_12	Copenhagen	Nasal Carriage	33	37	166688	316256	2684872	GAIIX
NC_CPH_13	Copenhagen	Nasal Carriage	80	25	280545	464198	2692431	GAIIX
NC_CPH_14	Copenhagen	Nasal Carriage	209	33	189900	468662	2751516	GAIIX
NC_CPH_15	Copenhagen	Nasal Carriage	167	45	167277	461895	2716660	GAIIX
NC_CPH_16	Copenhagen	Nasal Carriage	184	79	171232	646362	2658701	GAIIX
NC_CPH_17	Copenhagen	Nasal Carriage	112	28	215791	385824	2753248	GAIIX

NC_CPH_18	Copenhagen	Nasal Carriage	783	54	184466	482135	2716693	GAIIX
NC_CPH_19	Copenhagen	Nasal Carriage	68	32	162821	722131	2719857	GAIIX
NC_CPH_1	Copenhagen	Nasal Carriage	178	47	343309	655204	2697596	GAIIX
NC_CPH_20	Copenhagen	Nasal Carriage	74	29	351183	665424	2787625	MiSeq
NC_CPH_21	Copenhagen	Nasal Carriage	49	36	194808	646889	2753512	GAIIX
NC_CPH_22	Copenhagen	Nasal Carriage	199	67	206154	468694	2676592	GAIIX
NC_CPH_23	Copenhagen	Nasal Carriage	52	41	158856	468701	2654246	GAIIX
NC_CPH_24	Copenhagen	Nasal Carriage	95	46	162607	664666	2743039	GAIIX
NC_CPH_25	Copenhagen	Nasal Carriage	34	33	214263	494723	2671877	GAIIX
NC_CPH_2	Copenhagen	Nasal Carriage	181	37	329760	1155853	2736412	GAIIX
NC_CPH_3	Copenhagen	Nasal Carriage	40	31	184575	313187	2694169	GAIIX
NC_CPH_4	Copenhagen	Nasal Carriage	98	40	183969	574433	2694313	GAIIX
NC_CPH_5	Copenhagen	Nasal Carriage	90	44	185615	494695	2712613	GAIIX
NC_CPH_6	Copenhagen	Nasal Carriage	107	50	193286	468794	2734055	GAIIX
NC_CPH_7	Copenhagen	Nasal Carriage	342	62	158303	469018	2731314	GAIIX
NC_CPH_8	Copenhagen	Nasal Carriage	191	51	283074	495255	2697599	GAIIX
NC_CPH_9	Copenhagen	Nasal Carriage	431	63	172019	494602	2670614	GAIIX
SAB09_AKH_10	Aarhus	Bacteremia	79	45	136137	313407	2723926	GAIIX
SAB09_AKH_11	Aarhus	Bacteremia	114	47	158229	457865	2677592	GAIIX
SAB09_AKH_12	Aarhus	Bacteremia	133	47	169092	313291	2745355	GAIIX
SAB09_AKH_13	Aarhus	Bacteremia	58	144	170256	908254	2769077	GAIIX
SAB09_AKH_14	Aarhus	Bacteremia	152	54	184676	431525	2787927	GAIIX
SAB09_AKH_15	Aarhus	Bacteremia	16	50	110813	255155	2709983	GAIIX
SAB09_AKH_16	Aarhus	Bacteremia	103	34	287183	468174	2688594	GAIIX
SAB09_AKH_17	Aarhus	Bacteremia	27	45	171718	494662	2732093	GAIIX
SAB09_AKH_18	Aarhus	Bacteremia	108	33	184630	494427	2692070	GAIIX
SAB09_AKH_19	Aarhus	Bacteremia	39	47	158439	722462	2715764	GAIIX
SAB09_AKH_1	Aarhus	Bacteremia	63	45	167306	495066	2788470	GAIIX
SAB09_AKH_20	Aarhus	Bacteremia	23	102	154951	327483	2794460	GAIIX
SAB09_AKH_21	Aarhus	Bacteremia	34	53	184343	460076	2755489	GAIIX
SAB09_AKH_22	Aarhus	Bacteremia	74	49	184460	468768	2709662	GAIIX
SAB09_AKH_23	Aarhus	Bacteremia	26	41	194448	442472	2730410	GAIIX
SAB09_AKH24	Aarhus	Bacteremia	131	217	147925	338218	2810339	MiSeq
SAB09_AKH25	Aarhus	Bacteremia	293	107	326537	423024	2779042	MiSeq
SAB09_AKH_2	Aarhus	Bacteremia	105	38	200824	779139	2730885	GAIIX
SAB09_AKH_3	Aarhus	Bacteremia	121	49	127213	254263	2726969	GAIIX
SAB09_AKH_4	Aarhus	Bacteremia	164	71	171887	493631	2690786	GAIIX
SAB09_AKH_5	Aarhus	Bacteremia	133	32	184600	494587	2753342	GAIIX
SAB09_AKH_6	Aarhus	Bacteremia	205	56	158349	494618	2775870	GAIIX
SAB09_AKH_7	Aarhus	Bacteremia	80	48	330493	680527	2759742	MiSeq
SAB09_AKH_8	Aarhus	Bacteremia	124	33	170268	313350	2737682	GAIIX
SAB09_AKH_9	Aarhus	Bacteremia	36	31	206832	338956	2692074	GAIIX
SAB09_CPH_10	Copenhagen	Bacteremia	87	33	209320	691461	2703100	GAIIX

SAB09_CPH_11	Copenhagen	Bacteremia	53	39	158177	313364	2736996	GAIIX
SAB09_CPH_12	Copenhagen	Bacteremia	21	50	181077	657000	2702137	GAIIX
SAB09_CPH_13	Copenhagen	Bacteremia	33	39	195336	458973	2692455	GAIIX
SAB09_CPH_14	Copenhagen	Bacteremia	19	43	126109	393589	2729746	GAIIX
SAB09_CPH_15	Copenhagen	Bacteremia	35	39	184044	576716	2736790	GAIIX
SAB09_CPH_16	Copenhagen	Bacteremia	106	40	158299	494297	2723790	GAIIX
SAB09_CPH_17	Copenhagen	Bacteremia	97	99	213963	370378	2722638	GAIIX
SAB09_CPH_18	Copenhagen	Bacteremia	91	39	183928	467556	2632418	GAIIX
SAB09_CPH_19	Copenhagen	Bacteremia	128	92	257154	568428	2757417	MiSeq
SAB09_CPH_1	Copenhagen	Bacteremia	132	84	184633	468696	2702790	GAIIX
SAB09_CPH_20	Copenhagen	Bacteremia	130	34	183949	494645	2749239	GAIIX
SAB09_CPH_21	Copenhagen	Bacteremia	25	46	158297	461224	2715539	GAIIX
SAB09_CPH_22	Copenhagen	Bacteremia	82	30	162373	468753	2704088	GAIIX
SAB09_CPH_23	Copenhagen	Bacteremia	107	35	176482	369325	2694842	GAIIX
SAB09_CPH25	Copenhagen	Bacteremia	124	117	139821	405356	2745692	MiSeq
SAB09_CPH_2	Copenhagen	Bacteremia	177	40	184487	313317	2709229	GAIIX
SAB09_CPH_3	Copenhagen	Bacteremia	112	39	168720	938064	2688648	GAIIX
SAB09_CPH_4	Copenhagen	Bacteremia	43	56	249612	445173	2799171	GAIIX
SAB09_CPH_5	Copenhagen	Bacteremia	18	64	150121	312836	2731883	GAIIX
SAB09_CPH_6	Copenhagen	Bacteremia	1587	60	171034	468894	2702652	GAIIX
SAB09_CPH_7	Copenhagen	Bacteremia	17	68	140404	547730	2705558	GAIIX
SAB09_CPH_8	Copenhagen	Bacteremia	52	96	128043	459530	2734897	GAIIX
SAB09_CPH_9	Copenhagen	Bacteremia	38	98	169554	386368	2779611	GAIIX
SAB09_t004_2	Copenhagen	Bacteremia	56	36	142085	895468	2745827	GAIIX

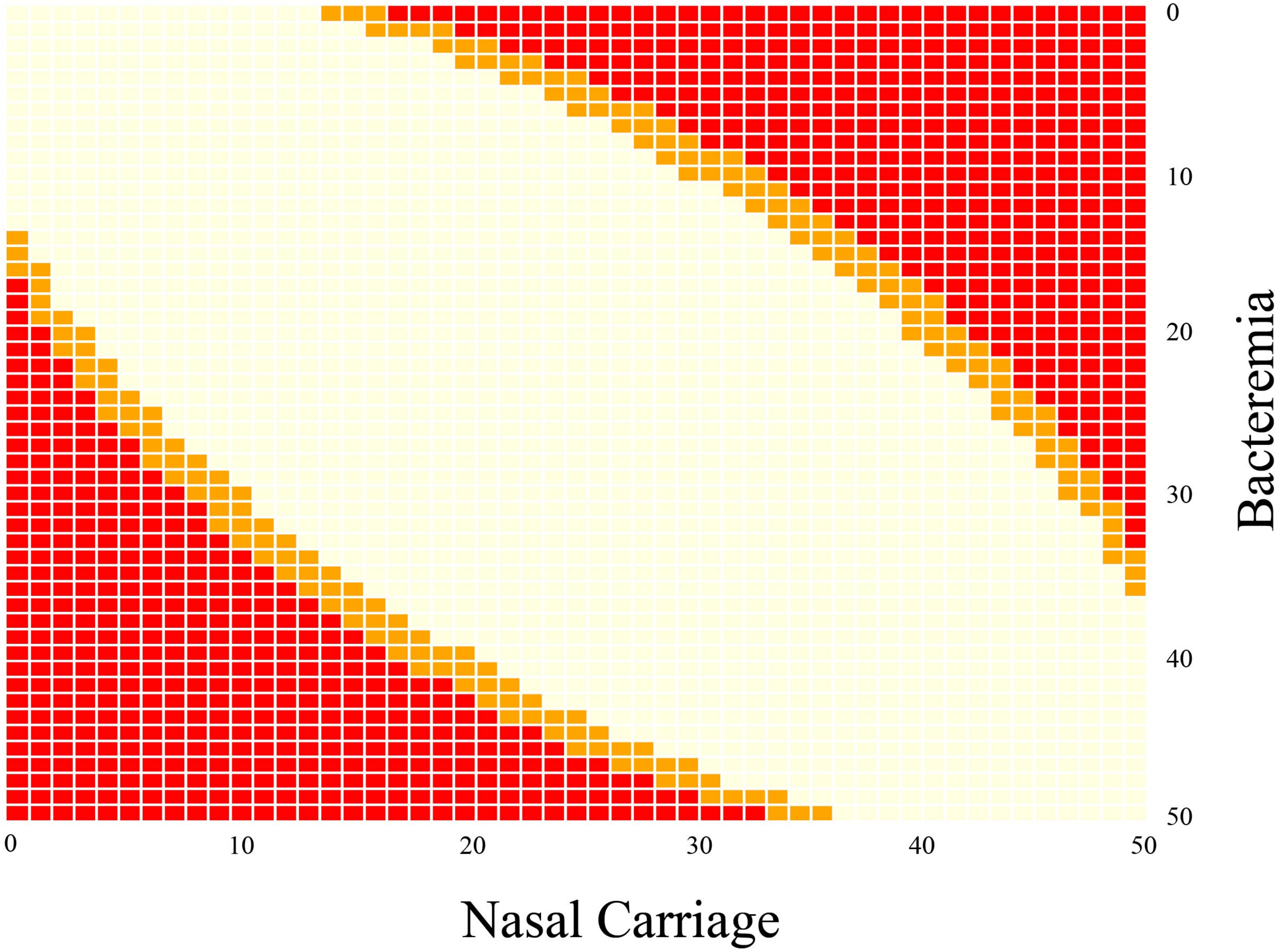

Supplemental Figure S1. Powerplot demonstrating the number of NC or SAB-only isolates that must contain a particular SNP for a Fisher's exact test to be significant after multiple testing correction using Bonferroni. Individual squares represent single SNPs present in X-number of NC and Y-number of SAB isolates. For instance, the top right square indicates that SNP is present only in one SAB isolate and all 50 NC isolates. Significance level of SNPs are denoted by color; red squares represent a p-value <0.05 after multiple testing correction, orange squares represent a p-value >0.05 and < 1 after correction, while light yellow squares correspond to a p-value = 1 after correction.

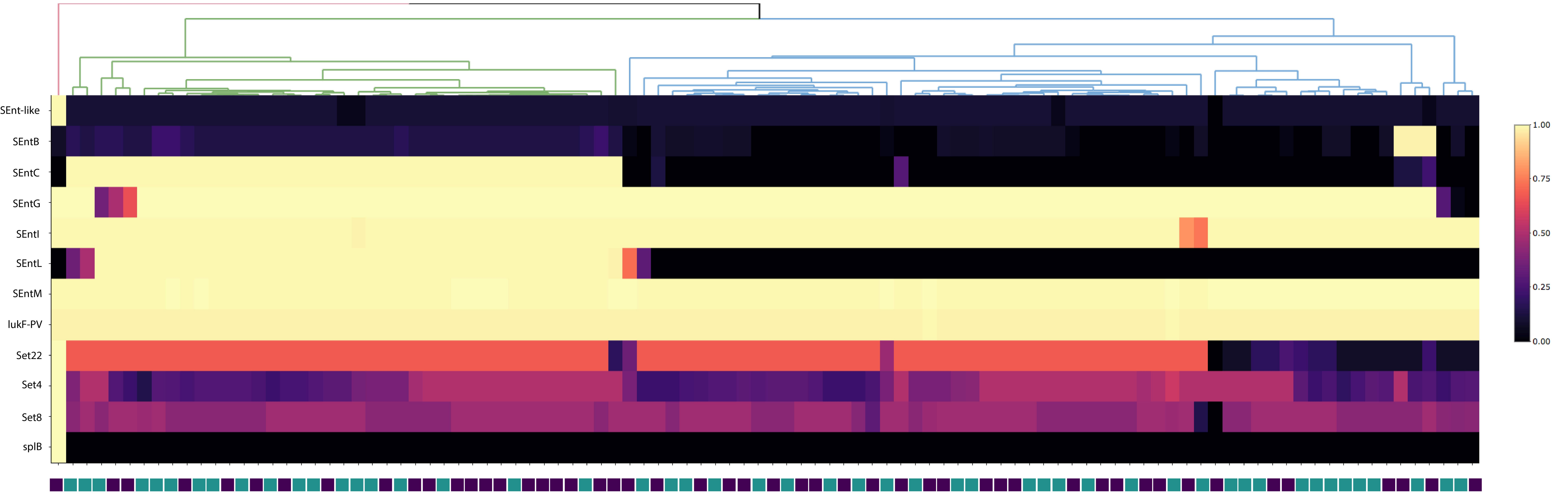

Supplemental Figure S2. Heat map showing the presence/absence of variable virulence genes of the two source types, SAB and NC. Light yellow represents presence of the gene whereas black represents absence (<20% of gene is present). Nasal carriage samples are denoted by purple squares and bacteremia samples are represented by green squares. Samples were clustered hierarchically based on gene content. No cluster of isolates by infection type was observed, therefore we found no association between the virulence genes screened and the infection type.
